# Targeting cell death in Crohn’s disease: from mechanisms to medicines

**DOI:** 10.1038/s41420-026-03005-1

**Published:** 2026-03-10

**Authors:** Ying Zhang, Yifan Zhou, Junyan Gao, Jiahui Jia, Xuanming Fan, Miao He, Zixuan He, Yu Bai

**Affiliations:** 1 Department of Gastroenterology, Changhai Hospital, Naval Medical University, Shanghai, China; 2 Department of General Practice, Changhai Hospital, Naval Medical University, Shanghai, China; 3 Department of Pharmacy, Changhai Hospital, Naval Medical University, Shanghai, China; 4 Changhai Clinical Research Unit, Changhai Hospital, Naval Medical University, Shanghai, China; 5 National key laboratory of Immunity and inflammation, Naval Medical University, Shanghai, China

**Keywords:** Cell death, Drug discovery

## Abstract

Crohn’s disease (CD) is a chronic inflammatory granulomatous disease that can damage the gastrointestinal tract. Existing treatment methods often fail to achieve satisfactory clinical effects. Although the pathogenesis of CD has not been fully elucidated, increasing evidence suggests that programmed cell death plays a key role in disease progression. This article comprehensively reviews 12 different mechanisms of cell death related to the pathogenesis of CD: apoptosis, necroptosis, pyroptosis, parthanatos, ferroptosis, autophagy-dependent cell death, cuproptosis, oxeiptosis, entotic cell death, netotic cell death, lysosome-dependent cell death, and alkaliptosis. On this basis, the article discusses targeted therapeutic strategies to regulate these cell death pathways, with a particular emphasis on their translational potential in clinical applications. Our analysis of existing studies concludes that autophagy, regulated by ATGs, is crucial in CD, and its dysregulation is associated with genetic factors (ATG16L1, IRGM, NOD2) and the mTOR signaling pathway, suggesting that autophagy may serve as a therapeutic target. Apoptosis and pyroptosis mediated by caspases and gasdermin, respectively, as well as ferroptosis and copper death, are all involved in the pathogenesis of CD, highlighting potential therapeutic strategies through the regulation of these pathways and cell death mechanisms (Graphical abstract 1).

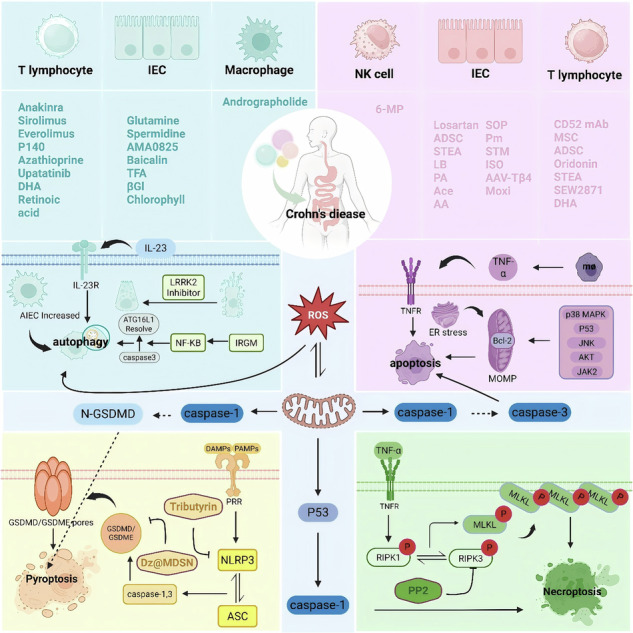

## Facts

- Multiple programmed cell death pathways are dysregulated in Crohn’s disease (CD), including apoptosis, necroptosis, pyroptosis, ferroptosis, cuproptosis, and autophagy.
- Key autophagy-related genes (e.g., ATG16L1, IRGM, NOD2) and signaling pathways (e.g., mTOR, LRRK2/NF-κB) are implicated in CD pathogenesis, influencing intestinal inflammation, barrier integrity, and bacterial clearance. Apoptosis in CD is regulated via both intrinsic (e.g., Bcl-2 family, mitochondrial ROS/JNK) and extrinsic (e.g., TNF-α/TNFR) pathways, with network-like dysregulation involving molecules like Runx2, VPS4B, circSMAD4, and MPST.
- Necroptosis occurs in CD patients, driven by RIPK3/MLKL activation, and can be modulated by factors like ATG16L1 and IFN-λ. Pyroptosis contributes to CD pathology through GSDMD/GSDME cleavage, with regulatory roles for NLRP3, IRGM, and specific circRNAs (e.g., circGMCL1, circPRKAR1B).
- Ferroptosis is characterized by GPX4 suppression and lipid peroxidation in CD, exacerbated by factors like adherent-invasive E. coli (AIEC) and pro-inflammatory diets.
- Several pharmacological agents and natural compounds (e.g., rapamycin, losartan, sirolimus, and andrographolide) show efficacy in experimental CD models by modulating specific cell death pathways.

## Open questions

- What are the mechanistic roles of dysregulated cell death in driving anti-TNF and other biologic therapy resistance in CD, and can modulating these pathways reverse resistance?
- Could combination therapies simultaneously targeting multiple cell death pathways offer synergistic benefits, and how can their safety and efficacy be optimally evaluated?
- How do gut microbiota, particularly pathobionts like AIEC, mechanistically influence host cell death decisions to promote CD initiation and progression?
- What are the specific roles and clinical-translational potential of non-coding RNAs (e.g., circRNAs, miRNAs) within the regulatory networks of cell death in CD?
- Can robust biomarkers derived from cell death pathways be developed and validated for early diagnosis, disease stratification, and prognosis prediction in CD patients?

## Introduction

Inflammatory bowel disease (IBD) is a chronic inflammatory disorder of the gastrointestinal tract, classified into Crohn’s disease (CD), ulcerative colitis (UC), and indeterminate colitis (IC). Since the early 20th century, the incidence of IBD has risen markedly in industrialized countries and emerging economies, with the rise in CD incidence being particularly prominent [1]. It predominantly affects the terminal ileum and cecum, but can also involve the entire gastrointestinal tract. This involvement leads to acute or chronic necrosis and fibrosis. Initially, most patients develop stomachache, diarrhea, and weight loss, and approximately half of the patients develop complications such as intestinal strictures, fistulas, or abscesses as the disease progresses. Current therapeutic drugs, including 5-aminosalicylic acid, glucocorticoids, immunosuppressants, and biological agents, focus on preventing complications and halting disease progression by achieving deep and sustained remission. However, these therapies generally have limitations such as long treatment cycles, significant adverse reactions, high costs, and low response rates in some patients [2]. Therefore, exploring new and efficient treatment strategies for CD has become a medical priority that needs an urgent breakthrough.

Markedly increased studies demonstrate multiple cell death pathways arise in CD patients and experimental models [3]. Programmed cell death (PCD) is an evolutionarily conserved mechanism maintaining physiological homeostasis, organismal development, and defense against disease [4]. CD risk alleles such as NOD2, ATG16L1, LRRK2, IRGM, IL23R, and STAT3 demonstrated dysregulated cell death in CD progression [5]. Emerging evidence suggests that agents targeting cell death have considerable therapeutic potential in CD management, including apoptosis, necroptosis, pyroptosis, parthanatos, ferroptosis, autophagy-dependent cell death, cuproptosis, oxeiptosis, entotic cell death, netotic cell death, lysosome-dependent cell death, and alkaliptosis. Thus, we systematically review the cell death mechanism implicated in CD and investigate agents or drugs directed at cell death as potential strategies for CD treatment.

## Mechanisms of cell death induced by CD

### Autophagy in CD (Fig. 1)

Autophagy is regulated by autophagy-related genes (ATGs), divided into microautophagy, chaperone-mediated autophagy (CMA), and macroautophagy [6]. While microautophagy is predominantly observed in yeast, this review focuses on macroautophagy and CMA.

**Fig. 1:**
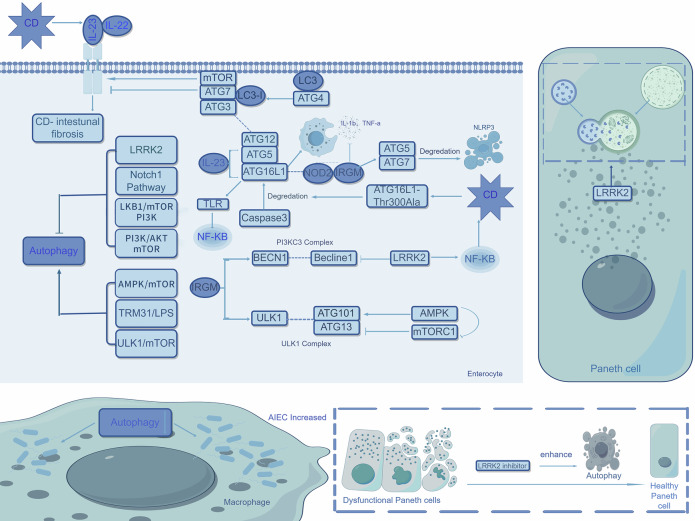
Autophagy in CD. Current studies have identified seven major pathways involved in the regulation of autophagy. Among these, activation of the AMPK/mTOR, TRM31/LPS, and ULK1/mTOR pathways promotes autophagy, whereas activation of the LRRK2 (specifically in Paneth cells), Notch1, LKB1/mTOR/PI3K, and PI3K/AKT/mTOR pathways exerts inhibitory effects. In CD patients, elevated levels of IL-23 and IL-22 are observed in affected tissues, with mTOR signaling enhancing their pro-inflammatory effects. In contrast, the ATG7-associated pathway counteracts these effects. Mechanistically, IL-23 facilitates autophagy through ATG5 and ATG16L1 while simultaneously promoting macrophage secretion of IL-1β and TNF-α. This cytokine further amplifies NF-κB-dependent inflammatory signaling via TLR pathways.ATG16L1 critically mediates the translocation of the ATG12-ATG5-LC3 complex to autophagosome membranes, thereby driving autophagosome formation. The IRGM protein coordinates antimicrobial and anti-inflammatory responses through interactions with NOD2 and ATG16L1. Notably, IRGM fails to mediate NLRP3 degradation under conditions of ATG5 or ATG7 deficiency. In CD patients, the ATG16L1-Thr300Ala mutation enhances Caspase-3-mediated degradation of ATG16L1. IRGM additionally regulates autophagic initiation complex assembly via direct interactions with ULK1 and BECN1. Pathologically, CD patient-derived MDMs exhibit increased survival of AIEC. Importantly, pharmacological inhibition of LRRK2 in dysfunctional Paneth cells restores autophagic flux and improves cellular functionality.

The activation of the UNC-51-like kinase 1 (ULK1) complex initiates macroautophagy. When nutrition is sufficient, mTOR inhibits ULK1-2, thereby suppressing autophagy. In contrast, stress signals activate 5′-AMP-activated protein kinase (AMPK), which in turn stimulates vacuolar protein sorting 34 (VPS34). The VPS34 complex helps phosphatidylinositol-3-kinase (PI3K) generate phosphatidylinositol-3-phosphate (PI3P). PI3P recruits PI3P-binding molecules forming pre-autophagosomal structure (PAS), also termed the phagophore. LC3 is cleaved into LC3-I, which conjugates with phosphatidylethanolamine via ubiquitin-like modification, forming LC3-II. LC3-II anchors in the membrane of the expanding phagophore. The phagophore elongates and ultimately seals, forming a double-membraned autophagosome. Autophagosome is transported to the perinuclear region and fuses with proximal lysosomes, generating autolysosomes [7]. Finally, lysosomal hydrolases degrade the sequestered cargo for nutrient recycling.

When CMA occurs, proteins with a specific pentapeptide motif (Lys-Phe-Glu-Arg-Gln, KFERQ) are recognized and bound by the heat shock cognate protein 70, which carries selected proteins to the lysosomal membrane [8]. Next, lysosome-associated membrane protein-2A (lamp2a) recognized the chaperone-protein complex. Finally, the proteins are moved into the lysosomal lumen and degraded with the help of other chaperones, such as lysosomal (Lys)-heat shock cognate protein 70 [9].

Autophagy dysregulation in CD involves multiple genetic factors such as ATG16L1, IRGM, and NOD2, as well as critical signaling pathways, including mTOR and LRRK2/NF-κB. It also includes pathogenic agents like adherent-invasive Escherichia coli (AIEC) and regulatory mechanisms such as RNA-mediated regulation and post-translational modifications. These factors collectively drive intestinal inflammation, fibrosis, impaired bacterial clearance, and compromised epithelial barrier function, emphasizing autophagy in CD management.

#### ATG16L1

ATG16L1 facilitates the transfer of the ATG12-ATG5-LC3 complex to autophagosome membranes, thereby assisting autophagosome formation. It regulates intestinal homeostasis by interacting with RICK/RIP2 through restraining Toll-like receptor (TLR)-mediated pro-inflammatory cytokine responses [10]. Notably, the ATG16L1-Thr300Ala mutant exhibits significant upregulation in CD. The mutant disrupts autophagic flux by promoting caspase-3-mediated degradation of ATG16L1, which may worsen intestinal barrier injury by reducing the ability of intestinal epithelial cells to clear intracellular pathogens [11–19]. Furthermore, it promotes the release of pro-inflammatory cytokines (IL-1β and TNF-α) and establishes a chronic inflammatory microenvironment by activating the TLR-mediated NF-κB pathway. Collectively, these mechanisms suggest that ATG16L1 may be a key target for CD treatment.

#### IRGM

IRGM manages autophagy initiation complex formation by interacting with ULK1 and BECN1. It exerts antibacterial and anti-inflammatory effects by associating with autophagy-related proteins NOD2 and ATG16L1. Deficiency of IRGM1 expression significantly enhances susceptibility to chemically induced colitis, partly due to increased accessibility of commensal bacteria to intestinal tissues. IRGM mediates the autophagic degradation of NLRP3 inflammasome components. In the absence of essential autophagy proteins ATG5 or ATG7, IRGM may mediate autophagy in Crohn’s disease (CD) rather than promote NLRP3 degradation [20].

#### mTOR

mTOR, the mechanistic target of rapamycin, regulates cellular growth and metabolism through two complexes: mTORC1 and mTORC2. It promotes anabolic processes, including ribosome biogenesis, protein synthesis, nucleotide production, and lipid biosynthesis, as well as suppresses catabolic processes like autophagy. While current research predominantly focuses on mTORC1, mTORC2 deficiency could impair goblet cell mucin secretion via mTORC2-mediated FoxO1 to exacerbate microbial dysbiosis. In CD, the AMPK/mTOR signaling axis governs autophagy induction and contributes to intestinal fibrosis [21, 22]. Studies in CD mouse models have shown that suppressing the mTOR/ULK1 pathway activated autophagy [23]; inhibition of mTOR with rapamycin or gene knockout suppressed IL-23 expression, reduced intestinal IL-22 production, and ameliorated fibrosis [24]. In the IL-10-deficient CD mouse model, autophagy is amplified through suppressing the PI3K/Akt/mTOR axis [25, 26], while activation of this pathway attenuated CD symptoms in IL-10 knockout mice[27]. While basal autophagy maintains cellular homeostasis, its excessive activation may deplete intestinal epithelial stem cells. In CD rat models, the signaling network of LKB1-mTOR-PI3KC mitigates intestinal inflammation through suppressing hyperactivated autophagy and modulating immune-related factor expression [28]. Thus, targeted modulation of mTOR signaling holds therapeutic potential to ameliorate disease progression.

#### LRRK2

Paneth cells provide a niche factor for neighboring intestinal stem cells by secreting antimicrobial peptides. Their dysfunction is implicated in inflammatory bowel disorders and is hypothesized to represent a potential origin of CD and UC. LRRK2 regulates macroautophagy catabolic processes and modulates lysozyme sorting in Paneth cells. LRRK2 inhibitors ameliorated CD progression in patients by enhancing autophagy to restore Paneth cell functionality [29]. Both CD patients and mice carrying the LRRK2 risk allele exhibited Paneth cell defects, including a reduction in the number of ileal Paneth cells and percentages of normal cells. Notably, in Lrrk2 G2019S knock-in mice, LRRK2 kinase inhibitors rescue these defects [29]. Membrane-associated LRRK2 stimulates Beclin-1 and suppresses autophagy by interacting with TAB2. In Beclin1-deficient HCT116 colonic epithelial cells, the expression level of LRRK2 is elevated, suggesting that autophagy inhibition may upregulate LRRK2. Furthermore, the severity of colitis is driven by LRRK2-mediated activation of components in the NF-κB pathway [30]. Inhibition of LRRK2 kinase activity induces macroautophagy with the help of PI3P/Beclin-1 independent of the mTOR/ULK1 axis [31]. LRRK2 may confer clinical benefits to CD patients by improving Paneth cell function and attenuating intestinal inflammation.

#### NOD2

Among the genetic risk factors for CD, polymorphisms in the nucleotide-binding oligomerization domain-containing protein 2 (NOD2) gene are currently recognized as the most significant [30]. The amino acid variants R702W, G908R, and frameshift mutation L1007fs are the most common polymorphisms of IBD in NOD2. NOD2 directly regulates selective autophagy of intracellular bacteria by recruiting ATG16L1 to the plasma membrane. ATG16L1 promoter hypermethylation is detected in intestinal organoids of NOD2 mutation carriers. Niemann–Pick disease type C1 (NPC1) mutations elevated the risk of severe early-onset CD by impairing autophagy [32, 33]. Autophagy activators such as rapamycin reverse the intracellular bacteria clearance deficiency induced by NOD2 mutation [34]. Furthermore, NOD2 invovled in mitochondrial ROS production controlled by autophagy. In CD patients with NOD2 mutations, atypical inflammatory phenotypes is drived by type I interferon responses via the cGAS-STING pathway. Additionally, it is unexplored whether the intestinal virome modulates autophagy via NOD2. In conclusion, as a key molecule in the pathogenesis of CD, NOD2 represents an important target for CD treatment.

#### AIEC

The function of AIEC replication is impaired in macrophages (MDMs) of CD patients. AIEC bacteria survival increased in CD patient MDMs relying on gene polymorphisms of IRGM, XBP-1, and ULK1 induced by CD. Adjusting AIEC may be a new strategy for CD treatment [35–39].

#### post-translational modification

Multiple post-translational modifications (PTMs) are involved in the interaction between CD and autophagy. CLEC12A, an early bacterial-associated sensor, initiates antibacterial autophagy through the ubiquitin-NDP52 pathway [40]. O-GlcNAc modification increases in CD provoking intestinal inflammation by NF-KB activation and autophagy suppression [41]. SUMOylation subverts host cell autophagy by AICE [42]. O-GlcNAcylation and SUMOylation may engage in crosstalk through shared substrates to impair autophagy-related proteins functionality. PTMs necessitate more investigations to concurrently address target specificity and global metabolic consequences, which simultaneously unveil novel scientific perspectives for personalized therapeutic strategies.

#### RNA

Circular RNAs (circRNAs) are closely related to CD pathogenesis. In peripheral blood mononuclear cells (PBMCs) of CD patients, hsa_circRNA_103124 expression upregulated significantly, which inhibites aotophagy [43, 44]. METTL3-mediated m^6^A modification of circPRKAR1B aggravated CD by weighting pyroptosis via colonic epithelial autophagy impairment [45].

Beyond circRNAs, microRNAs (miRNAs) also play pivotal roles in CD pathophysiology [46]. Lin et al. revealed that miR-143 may induce intestinal inflammation via ATG2B-mediated autophagy, while miR-130a-3p promotes CD progression through the ATG16L1/NF-KB pathway [47]. miR-376a-3p overexpression and miR-20a-5p downregulation influence autophagy-related substrates and systemic pro-inflammatory cytokine levels [48]. miR-874-3p directly suppressses ATG16L1 expression. A miR-874-3p mimic aberrantly modulates autophagy through LC3-II reduction in vitro model of CD [49]. Autophagy disruption influences CD by increasing Argonaute 2 (AGO2) levels to regulate differential miRNA expression [50]. circGMCL1 alleviates epithelial pyroptosis and preserves intestinal barrier function in CD by promoting autophagy through sponging miR-124-3p to regulate ANXA7 [51]. Exosomes secreted by intestinal epithelial cells (IECs) contain specific miRNAs post-AIEC infection, which transfer to recipient IECs, suppressing autophagy-mediated intracellular AIEC clearance [52].

The multidimensional mechanisms by which non-coding RNAs (circRNAs and miRNAs) influence CD progression through autophagy regulation and inflammatory responses, highlighting the critical roles of epitranscriptomic modifications (m^6^A), competing endogenous RNA (ceRNA) networks, and exosome-mediated intercellular communication. Future research transcends the traditional “one molecule-one phenotype” paradigm to investigate the dynamic interactome of non-coding RNAs and their multidimensional correlations with clinical features, while advancing RNA-based therapeutics to develop precision intervention strategies that overcome heterogeneity challenges in CD treatment.

**Fig. 2:**
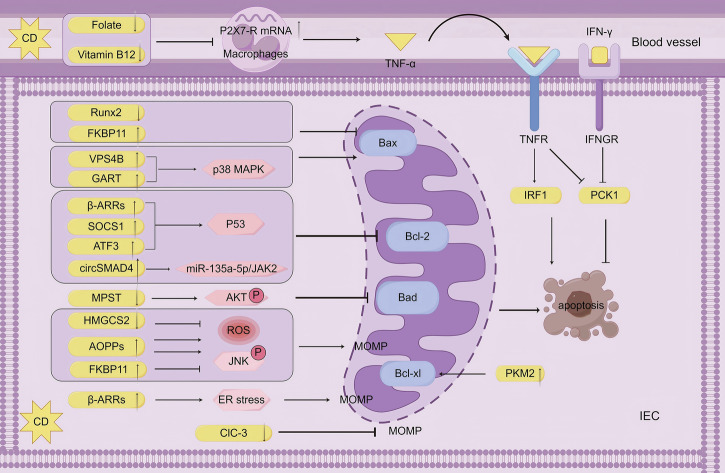
Apoptosis in CD. In CD, the apoptotic regulation pathway exhibits network-like dysregulation through multiple molecular mechanisms. At the intrinsic apoptosis level, pro-apoptotic pathways mainly involve Bax translocation, Bcl-2 inhibition, and TNF-α signaling activation; meanwhile, anti-apoptotic mechanisms rely on Bax inhibition, Bad signaling inhibition, and upregulation of anti-apoptotic proteins. Mitochondrial function is bidirectionally regulated by the reactive oxygen species (ROS)/JNK pathway and endoplasmic reticulum stress. For example, Runx2 downregulation and FKBP11 upregulation collaboratively inhibit apoptosis by suppressing Bax expression, whereas VPS4B and GART elevation promote Bax translocation via the p38 MAPK pathway, thereby promoting apoptosis. Additionally, β-adrenergic receptors (β-ARRs), SOCS1, and ATF3 inhibit Bcl-2 transcription through the p53 pathway, and circSMAD4 downregulates Bcl-2 expression via the miR-135a-5p/JAK2 pathway; these factors collectively promote apoptosis. MPST reduction inhibits Bad-mediated signaling through AKT phosphorylation, and PKM2 elevation suppresses apoptosis by enhancing Bcl-xL expression. At the extrinsic apoptosis level, reduced plasma folate and vitamin B12 inhibit TNF-α expression, while macrophage P2X7 receptor (P2X7-R) elevation enhances TNF-α expression, subsequently activating IRF1 and synergizing with IFN-γ to suppress PCK1, thereby promoting apoptosis. CIC-3 exerts anti-apoptotic effects by inhibiting mitochondrial outer membrane permeabilization (MOMP).

### Apoptosis in CD (Fig. 2)

Apoptosis, a tightly regulated form of programmed cell death, is mediated by caspases through the intrinsic (mitochondrial) and extrinsic (death receptor) pathways, thereby maintaining cellular homeostasis under both physiological and pathological conditions. Its hallmark morphological feature is the formation of membrane-bound apoptotic bodies.

The intrinsic apoptosis pathway is orchestrated by the Bcl-2 protein family, which comprises three functional subgroups: pro-apoptotic proteins (BAX, BAK, and BOK), anti-apoptotic proteins (BCL-2, BCL-XL, and MCL-1), and BH3-only proteins (BIM, PUMA, and tBID). Pro-apoptotic proteins induce mitochondrial outer membrane permeabilization (MOMP), facilitating the release of cytochrome c (Cyt C) and second mitochondria-derived activator of caspases (Smac) into the cytosol. Cyt C forms the apoptosome complex with apoptotic protease activating factor-1 (APAF-1), thereby activating caspase-9, which subsequently cleaves effector caspases (caspase-3, -7, and -6) to execute apoptosis. Smac promotes caspase-3 and -7 activation by neutralizing inhibitor of apoptosis proteins (IAPs), including cIAP-1, cIAP-2, and XIAP [53]. The c-Jun N-terminal kinase (JNK) pathway directly impairs mitochondrial membrane integrity. BH3-only proteins antagonize anti-apoptotic members to liberate pro-apoptotic effectors. Additionally, intracellular reactive oxygen species (ROS) accumulation and endoplasmic reticulum (ER) stress exacerbate MOMP.

Regulation of the Bcl-2 family involves multiple signaling pathways. The p38 mitogen-activated protein kinase (p38 MAPK) cascade mediates Bax translocation from the cytosol to the outer mitochondrial membrane (OMM). AKT signaling suppresses apoptosis either by directly phosphorylating Bad or by inhibiting Forkhead box O (FoxO), a transcription factor that promotes pro-apoptotic gene expression. Furthermore, p53 binds to the Bcl-2 promoter region to repress its transcription. Dysregulation of these pathways is implicated in CD.

The extrinsic apoptosis pathway is initiated by caspase-8 activation through ligand-death receptor interactions, including FASL-FAS, TNF-α-TNFR1, TL1A-DR3, and TRAIL-TRAILR1/2. Dysregulation of these pathways is implicated in the pathogenesis of CD, where excessive TNF-α signaling contributes to inflammation.

The intrinsic apoptosis pathway plays a pivotal role in CD pathogenesis through its regulation of Bcl-2 family protein expression and related gene networks. Notably, circSMAD4, a mitochondria-associated circular RNA, demonstrates significant upregulation in inflamed colonic tissues of CD patients. Mechanistically, circSMAD4 suppresses Bcl-2 expression via the miR-135a-5p/JAK2 signaling axis [54]. Furthermore, intestinal epithelial cells in CD patients exhibit marked overexpression of vacuolar protein sorting 4B (VPS4B) and glycinamide ribonucleotide formyltransferase (GART), both of which modulate apoptotic processes through p38 MAPK pathway activation [55, 56].

The 3-mercaptopyruvate sulfurtransferase (MPST) exhibits significant downregulation in colonic tissues of CD patients [57]. As a regulator of AKT phosphorylation, MPST deficiency may contribute to disease pathogenesis. Intestinal epithelial cells in CD demonstrate reduced expression of runt-related transcription factor-2 (Runx2), which normally suppresses BAX protein levels [58]. Concurrently, elevated pyruvate kinase M2 (PKM2) expression upregulates Bcl-xL [59], while mesenteric adipose tissue (MAT) and lamina propria lymphocytes (LPL) show characteristic apoptotic dysregulation with increased Bcl-2 and decreased BAX expression [60, 61].

The JNK signaling pathway plays a pivotal role in CD pathophysiology. Intestinal epithelial cells exhibit compartmentalized JNK regulation: FKBP11-mediated JNK suppression through BAX reduction promotes epithelial survival [62], whereas advanced oxidation protein product (AOPP)-induced ROS generation activates JNK phosphorylation [63]. This dichotomous regulation may underlie intestinal barrier dysfunction and chronic inflammation.

Mitochondrial dysfunction emerges as a central feature in CD. Reduced HMGCS2 expression elevates ROS production [64], suggesting therapeutic potential for ketogenic diets. Similarly, decreased ClC-3 expression enhances mitochondrial membrane permeability [65]. Inflamed intestinal tissues show upregulated β-arrestins (β-arrs), triggering ER stress [66]. Coordinated upregulation of β-arrs, suppressor of cytokine signaling 1 (SOCS1), and activating transcription factor 3 (ATF3) enhances p53 expression and activity [66–68].

These findings position mitochondrial pathways, particularly Bcl-2 family regulation, as promising therapeutic targets for CD management.

The extrinsic apoptosis pathway also plays a pivotal role in the pathogenesis of CD. TNF-α, a central regulator, initiates apoptotic signaling through binding to TNFR. Mechanistic studies reveal that TNF-α induces apoptosis via activation of interferon regulatory factor-1 (IRF1) [69]. Notably, elevated P2X7 receptor mRNA expression in inflamed intestinal epithelium and lamina propria of CD patients significantly amplifies TNF-α signaling [70]. Clinically, reduced plasma folate and vitamin B12 levels correlate with enhanced TNF-α expression in these patients [71]. Importantly, TNF-α synergizes with IFN-γ to downregulate phosphoenolpyruvate carboxykinase 1 (PCK1) expression [72]. Immunopathological investigations demonstrate that intestinal infiltration of M1 macrophages exacerbates disease progression through TNF-α-mediated apoptosis, leading to intestinal barrier disruption [73]. Contrastingly, intestinal epithelial autophagy exerts protective effects by counteracting TNF-induced apoptosis, highlighting its therapeutic potential [74]. The crosstalk between programmed cell death pathways may induce “self-healing” mechanisms, offering novel strategies to minimize therapeutic interventions and associated adverse effects, a promising avenue for future research.

Furthermore, study has found that catalase activity is significantly reduced in peripheral blood T cells of CD patients, leading to decreased T cell apoptosis [75]. This provides a new research direction for the treatment of CD. Apoptosis is regulated by intrinsic and extrinsic pathways mediated by caspases, with Bcl-2 family proteins playing a crucial role in the intrinsic pathway, influencing mitochondrial outer membrane permeabilization (MOMP) and cytochrome c release. Dysregulation of these pathways, particularly involving TNF-α signaling, is implicated in Crohn’s disease (CD) pathogenesis, with notable alterations in Bcl-2 expression, JNK signaling, and mitochondrial dysfunction highlighting potential therapeutic targets.

**Fig. 3:**
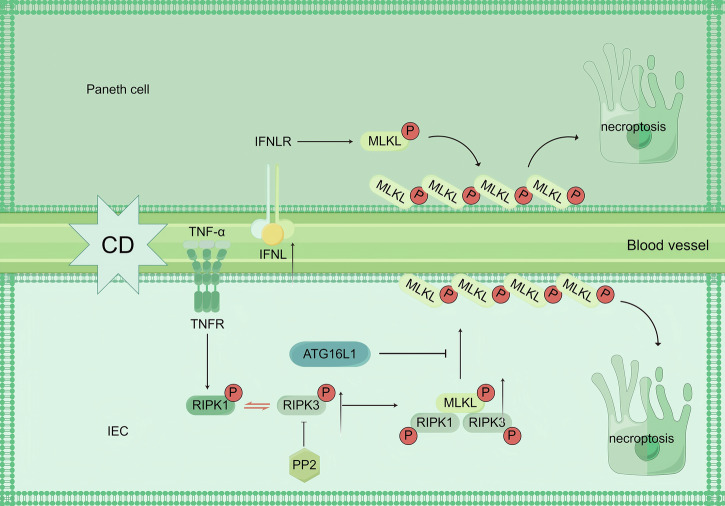
Necroptosis in CD. In CD, the necroptosis pathway is regulated by multiple factors: on one hand, ATG16L1 inhibits TNF-α-mediated necroptosis by preventing MLKL oligomerization in intestinal epithelial cells; on the other hand, IFN-λ promotes this process by activating MLKL to trigger a cascade reaction. Specifically, highly expressed IFN-λ in the serum and ileal tissues of CD patients activates MLKL in Paneth cells, thereby initiating necroptosis. PP2 inhibits MLKL phosphorylation and oligomerization by disrupting RIPK3 oligomerization, ultimately blocking the necroptosis pathway in intestinal epithelial cells.

### Necroptosis in CD (Fig. 3)

Necroptosis represents a lytic form of programmed cell death that is typically initiated when apoptotic pathways are compromised. This process can be triggered either through extracellular ligand-death receptor interactions (e.g., TNF-TNFR1 or FAS-FASL) or via intracellular signaling pathway activation. Under conditions of caspase-8 inhibition or functional insufficiency, receptor engagement leads to the sequential activation of two pivotal kinases: RIPK1 and RIPK3. These kinases form a necrosome complex that phosphorylates the mixed lineage kinase domain-like (MLKL) protein, resulting in oligomerization and plasma membrane pore formation. Subsequent osmotic imbalance induces cell swelling and eventual membrane rupture, culminating in the characteristic lytic cell death.

Notably, necroptosis can also be initiated independently of death receptors through pattern recognition receptor signaling. Specifically, Toll-like receptor (TLR) engagement by pathogen-associated molecular patterns (PAMPs) activates RIPK3 via the adapter proteins TRIF or ZBP1, thereby bypassing RIPK1 to directly promote MLKL phosphorylation and execute the necroptotic death program.

Emerging evidence implicates necroptosis in CD pathogenesis. Günther et al. provided the first demonstration of necroptosis occurrence in the terminal ileum of CD patients [76]. Subsequent studies have revealed elevated expressions of RIPK3 and MLKL in inflamed intestinal tissues, with their activation shown to promote intestinal inflammation [77, 78]. These findings collectively establish that necroptosis exacerbates inflammatory responses through RIPK3/MLKL signaling.

Mechanistic studies in murine models reveal that ATG16L1 plays a protective role by maintaining intestinal barrier integrity through inhibition of RIPK3/MLKL complex formation, thereby attenuating CD progression [16]. Notably, elevated IFN-λ levels detected in CD patient serum and differentiated Paneth cells have been shown to activate MLKL [79], suggesting an additional regulatory mechanism.

Therapeutic potential exists in targeting necroptosis markers (RIPK1, RIPK3, and MLKL) and IFN-λ signaling. Particularly promising is RIPK1, which represents a shared node between necroptosis and apoptotic pathways, warranting further investigation as a potential therapeutic target for CD management.

**Fig. 4:**
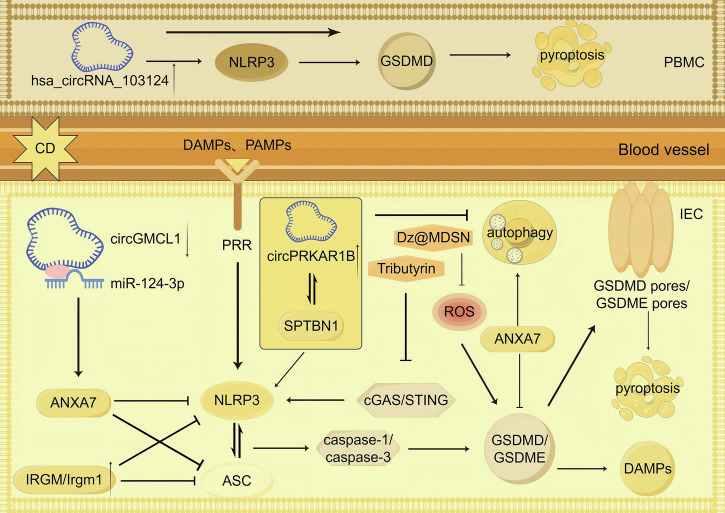
Pyroptosis in CD. In CD, pyroptosis is precisely regulated by multiple pathways: in IECs, PAMPs/DAMPs inhibit NLRP3 and ASC oligomerization through upregulation of IRGM and mediate their autophagic degradation to suppress pyroptosis; circGMCL1 enhances ANXA7 expression by sponging miR-124-3p to promote autophagy while reducing NLRP3, ASC and GSDMD expression to inhibit pyroptosis; whereas upregulated circPRKAR1B interacts with SPTBN1 to activate the NLRP3 inflammasome-mediated pyroptosis pathway and suppress autophagy. Dz@MDSN inhibits intestinal epithelial cell pyroptosis by scavenging ROS, while Tributyrin suppresses pyroptosis mediated by the cGAS-STING-NLRP3 pathway. In PBMCs, elevated expression of hsa_circRNA_103124 promotes pyroptosis by increasing NLRP3 and GSDMD levels.

### Pyroptosis in CD (Fig. 4)

Pyroptosis is characterized by gasdermin-mediated plasma membrane pore formation, primarily executed by gasdermin D (GSDMD) and gasdermin E (GSDME) proteins. The canonical pathway involves NOD-like receptor thermal protein domain-associated protein 3 (NLRP3) inflammasome assembly, where the adapter protein apoptosis-associated speck-like protein containing a caspase recruitment domain (ASC) and NIMA-related kinase 7 (NEK7) orchestrate caspase-1 activation. Active caspase-1 then proteolytically cleaves GSDMD, generating N-terminal fragments that oligomerize to form membrane pores, enabling the release of damage-associated molecular patterns (DAMPs) such as IL-1β and IL-18.

Intriguingly, under conditions of caspase-1 deficiency or pharmacological inhibition, an alternative pathway emerges: the inflammasome redirects its signaling to activate caspase-3, which subsequently processes GSDME to execute pyroptosis [80]. This mechanistic plasticity highlights the complexity of inflammatory cell death regulation in physiological and pathological contexts.

Emerging evidence demonstrates a critical involvement of pyroptosis in CD pathogenesis. Gao Tan and colleagues have elucidated that pyroptosis promotes CD progression through GSDME cleavage [81]. Notably, in peripheral blood mononuclear cells (PBMCs) from active CD patients, the upregulated hsa_circRNA_103124 enhances the expression of NLRP3 and GSDMD, key mediators of pyroptosis [82]. Additionally, in CD patients, both PAMPs and DAMPs serve dual functions by not only inducing pyroptosis but also upregulating immunity-related GTPase M (IRGM) expression to inhibit pyroptosis. Experimental studies using CD mouse models reveal that IRGM (Irgm1 in mice) exerts protective effects through multiple mechanisms: it disrupts NLRP3 and ASC oligomerization, inhibits inflammasome assembly, and mediates selective autophagic degradation of NLRP3 and ASC components [20].

Further mechanistic insights come from studies of circular RNAs (circRNAs) in CD. Intestinal epithelial cells from inflamed regions of CD patients exhibit significantly reduced circGMCL1 expression. In IL-10 knockout CD mouse models, circGMCL1 functions as a molecular sponge for miR-124-3p, leading to increased ANXA7 expression. This regulatory axis promotes autophagy while simultaneously suppressing the expression of pyroptosis-related proteins NLRP3, ASC, and GSDMD [51]. Conversely, in CD colon tissues, elevated levels of circPRKAR1B promote pyroptosis and inhibit autophagy through its interaction with the RNA-binding protein SPTBN1 [45]. These findings highlight the intricate crosstalk between pyroptosis and autophagy pathways in CD pathophysiology.

Therapeutic targeting of pyroptosis-related molecules, including NLRP3, caspase-1, GSDMD, and GSDME, represents a promising treatment approach for CD. Current evidence suggests that pharmacological modulation of these key mediators could potentially attenuate excessive inflammatory responses while preserving beneficial immune functions. Moreover, the delicate interplay between pyroptosis and autophagy pathways offers novel opportunities for developing balanced therapeutic strategies. By simultaneously regulating both cell death mechanisms, it may be possible to achieve more precise control over intestinal inflammation while minimizing potential side effects associated with complete pathway inhibition.

### Ferroptosis in CD

Ferroptosis is a distinct form of regulated cell death characterized by iron-dependent lipid peroxidation that causes direct plasma membrane damage. The process initiates with the incorporation of polyunsaturated fatty acids (PUFAs) into membrane phospholipids through the coordinated action of acyl-CoA synthetase long-chain family member 4 (ACSL4) and lysophosphatidylcholine acyltransferase 3 (LPCAT3). These PUFA-containing phospholipids then undergo peroxidation catalyzed by Fe²⁺ and lipoxygenases (LOXs), leading to the characteristic membrane rupture of ferroptosis.

This lethal process is negatively regulated by the glutathione peroxidase 4 (GPX4)-dependent pathway. The system xc⁻ transporter, composed of solute carrier family 7 member 11 (SLC7A11) and solute carrier family 3 member 2 (SLC3A2) subunits, mediates cystine/glutamate exchange to provide cysteine for glutathione (GSH) biosynthesis. The synthesized GSH serves as an essential cofactor that enhances GPX4 activity to reduce lipid hydroperoxides into nontoxic lipid alcohols, thereby effectively suppressing ferroptosis execution.

Ferroptosis has emerged as a significant contributor to CD pathogenesis. Single-cell RNA sequencing analyses have revealed distinct ferroptosis signatures in CD patients [83]. The therapeutic potential of ferroptosis inhibition is supported by studies demonstrating that ferrostatin-1, a specific ferroptosis inhibitor, ameliorates TNBS-induced colitis in experimental models [84].

Clinical investigations have identified an elevated abundance of Escherichia coli, particularly AIEC strains, in the gut microbiota of CD patients. AIEC infection of intestinal epithelial cells induces ferroptosis through characteristic chemical features: reducing GPX4 expression while increasing lipid peroxide (LPO) accumulation [85]. This microbial-host interaction suggests AIEC may promote CD susceptibility by triggering ferroptotic cell death.

Furthermore, pro-inflammatory dietary patterns contribute to ferroptosis susceptibility by downregulating GPX4 expression. This occurs through HSPA5 suppression mediated by endoplasmic reticulum stress [86]. These findings position GPX4 as both a central regulator of ferroptosis and a promising therapeutic target in CD. Consequently, dietary interventions and microbial modulation represent novel therapeutic avenues for CD management through targeted regulation of cell death pathways.

### Cuproptosis, dependent cell death, and other forms of cell death in CD

Cuproptosis relies on intracellular copper ions (Cu^2+^) accumulation. It combines with lipoylated components of the tricarboxylic acid (TCA) cycle during mitochondrial respiration. It induces lipoylated protein aggregation, inhibits iron-sulfur cluster protein functionality, and ultimately leads to cellular demise. Mitochondria regulate Cu^2+^ homeostasis [87]. Copper ionophores chelate and transport copper into cells. Cuproptosis may react on CD pathogenesis. Cuproptosis-related genes show differential expression between CD patients and healthy controls [87]. However, most copper-associated differentially expressed genes (CuDEGs) exhibit downregulation in CD specimens and demonstrate negative correlations with immune cell infiltration [88]. Current research on cuproptosis in CD remains limited, predominantly relying on bioinformatic predictions from public databases. Nevertheless, targeting cuproptosis pathways presents a novel therapeutic frontier for CD treatment.

Parthanatos is a poly (ADP-ribose) polymerase 1 (PARP1)-mediated programmed cell death. It is triggered via five key processes: DNA damage initiation, PARP1 overactivation, PAR polymer accumulation, NAD⁺/ATP exhaustion, and AIF nuclear translocation. Parthanatos has been implicated in the pathogenesis of CD, though mechanistic investigations remain scarce. In CD patients and chemically induced murine colitis models, stanniocalcin-1 (STC1) stimulates parthanatos via the STC1-PARP1-JNK pathway and the JAK pathway [89].

“Oxeiptosis”(oxygen-induced cell death) is a reactive oxygen species (ROS)-sensitive, caspase-independent, non-inflammatory cell death. Virus excites intracellular ROS accumulation, which triggers cell death by cellular ROS sensors (KEAP1), PGAM5 phosphorylation, and the pro-apoptotic factor AIFM1. In CD patients, ROS expression is significantly increased [90, 91]. It gives a new scheme for CD management.

**Fig. 5:**
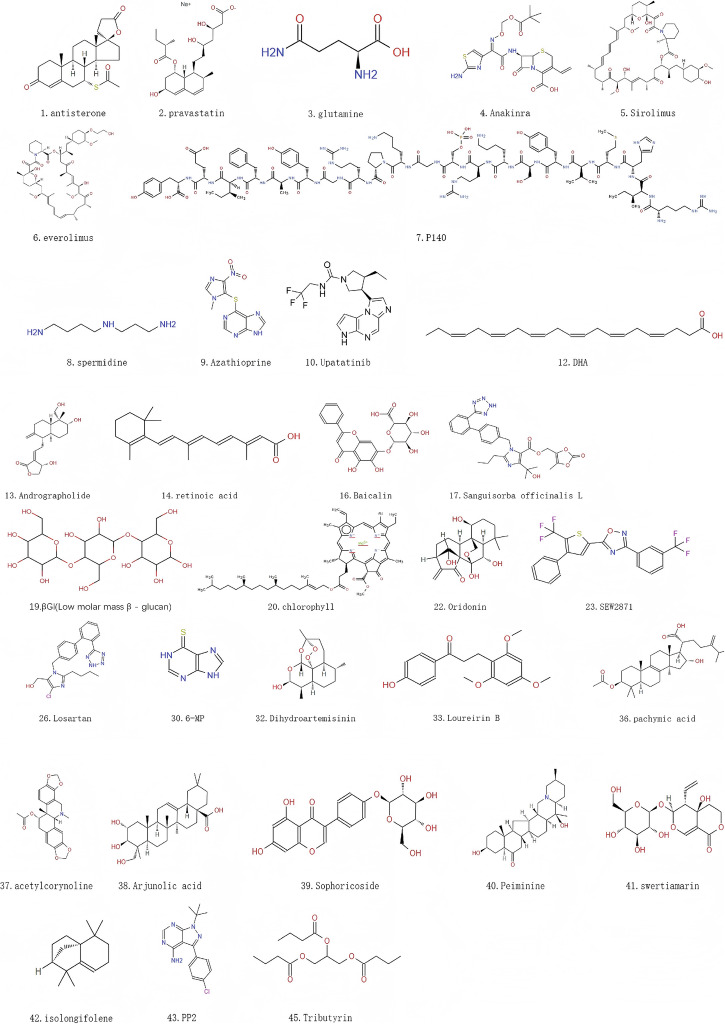
Chemical structures of agents for CD treatment. The serial numbers in the figure correspond to those in the summary table of drugs. Some of the drugs are cells, peptides, or composed of multiple substances; their corresponding chemical structure diagrams are not provided.

## Drugs/modulators targeting cell death in CD (Fig. 5 and Table 1)

### Drugs/modulators of anti-autophagy therapy of CD

Regulating autophagy could treat CD by multiple pathways, especially in immune cells and IECs, as shown in Table 1.

**Table 1:** Drugs/modulators targeting CD cell death.

Name	Category	Agents	Pre-Clinical Models	Relevant Effects or Mechanisms	Reference
autophagy					
antisterone	17 lactone steroids	Old drug	adult AIC (aAIC) zebrafish model	atg7-based autophagy activation	[138]
pravastatin	Competitive inhibitors of hydroxymethylglutarate monoacyl CoA reductase	Old drug	adult AIC (aAIC) zebrafish model	atg7-based autophagy activation	[138]
glutamine	amino acid	Biologics	Rat small intestine epithelial cell line (ATCC, Manassas, VA；CRL-1589) IEC-18	mTOR and p38 MAPK signaling pathways	[93]
Anakinra	Interleukin-1B receptor inhibitor	Old drug	Chronic granulomatous disease (CGD) mice and cells from patients with CGD	blocking IL1 restores defective autophagy in CGD	[101]
Sirolimus		Old drug	IBD children who were refractory to the conventional treatments	Inhibition of mTOR	[102]
	Macrolide immunosuppressants	Old drugs	IL-10 knockout mice	Inhibition of autophagy	[108]
P140	IPP-201101; sequence 131–151 of the U1-70K protein phosphorylated at Ser140	Biologics	Patients with moderately active Systemic lupus erythematosus (SLE)	Inhibit excessive activation of autophagy	[103]
spermidine	Natural polyamines in living organisms	Natural products	Senescence accelerated mouse-8 (SAMP8)	inhibition of mTORC1/activation of AMPK	[94–96]
everolimus		Old drug	IL-10 knockout mice	Inhibition of mTOR	[109]
Azathioprine	Imidazole derivatives of mercaptopurine	Old drugs	The HEK293 cells	Regulating mTORC1 signal transduction/stimulating UPR sensor PERK	[105]
Upatatinib	JAK1 receptor inhibitor	Old drugs	In vitro models of hepatic steatosis	Inhibit JAK1 and STAT3 phosphorylation, promote AMPK phosphorylated and autophagy markers	[106]
AMA0825	ROCK inhibitor	Synthetic compound	DSS induced colitis mouse model	Reduce MRTF and p38 MAPK activation	[97]
DHA	A long-chain polyunsaturated fatty acid	Natural products	IL-10 knockout mice	Inhibition of mTOR pathway triggers autophagy	[27]
Andrographolide	Diterpenoid compounds	Natural products	Mice with DSS-induced colitis	Inhibition STAT3 phosphorylation and P62 accumulation	[92]
retinoic acid		Old drugs	IL-10 knockout mice	Inhibition PI3K/AKT/mTOR signaling pathway	[25]
Xue-Jie-San		Natural products	CD-related rat intestinal fibrosis model	Blocking the Notch1 and FGL1 signaling pathways	[23]
Baicalin		Natural products	HT-29 cells exposed to lipopolysaccharide (LPS)	Regulating NF - κ B pathway	[98]
chlorophyll		Natural products	DSS-treated mice	Activate Akt and mTOR pathways	[100]
Sanguisorba officinalis L		Natural products	Mice with 2% dextran sodium sulfate (DSS)	Promote Atg7-dependent autophagy	[111]
Human bacterial total flavonoids (TFA)		Natural compound	A 2,4,6-Trinitrobenzenesulfonic acid (TNBS)-induced colitis model and IGF-1-treated intestinal fibroblasts	Regulate AMPK/mTOR signaling pathway	[22]
βGl(Low molar mass β - glucan)		Natural compound	TNBS-induced colitis model in Sprague-Dawley rats	Enhance autophagy	[99]
apoptosis					
CD52 mAb	Peptide	Old drug	IL-10 knockout mice	Inhibit TNF-α/TNFR2-mediated epithelial apoptosis	[112]
Oridonin	diterpene	Natural compound	TNBS-induced colitis mice	Upregulate the apoptosis of lymphocytes by inhibiting nuclear translocation of transcription factor nuclear factor-kappa B	[115]
SEW2871		Synthetic compound	IL-10 knockout mice	Reduce epithelial cell apoptosis by reducing TNF-α and IFN-γ levels	[117]
MSC	cell	Old drug	CD patients	Promote T cells apoptosis	[113]
Losartan		Old drug	TNBS-induced colitis mice	Inhibit epithelial apoptosis by increasing the B-cell lymphoma 2 (Bcl2)/Bcl-2-associated X protein (Bax) ratio and suppressing caspase-3 induction	[119]
STEA	Peptide	Natural compound	TNBS-induced colitis mice	Inhibit epithelial apoptosis	[116]
MF	multi-fibre mix	Natural compound	IL-10 knockout mice	Inhibit epithelial apoptosis	[131]
Tβ4	Peptide	Biologics	TNBS-induced colitis mice	Inhibit epithelial apoptosis by reducing IL-1β,TNF-α, and IL-10 levels	[130]
6-MP	purine	Old drug	CD patients	Promote NK cell apoptosis by inhibiting Rac1 activity	[129]
Moxi		Natural compound	TNBS-induced colitis mice	Inhibit TNF-α-mediated intestinal epithelial cells apoptosis	[118]
Dihydroartemisinin	Sesquiterpenes	Natural compound	TNBS-induced colitis mice	Promote activated CD4 + T lymphocytes apoptosis	[120]
Loureirin B	Chalcones	Natural compound	TNBS-induced colitis mice	Inhibit intestinal epithelial cells apoptosis by suppressing TNBS-induced the activation of IL-6/STAT3/NF-κB signaling pathway	[114]
ADSC	Cell	Old drug	TNBS-induced colitis mice	Inhibit intestinal epithelial cells apoptosis	[121]
pachymic acid	Triterpenes	Natural compound	TNBS-induced colitis mice	Inhibit intestinal epithelial cells apoptosis by inhibiting PI3K/AKT signaling	[122]
acetylcorynoline	Alkaloid	Natural compound	TNBS-induced colitis mice	Inhibit intestinal epithelial cells apoptosis by inhibiting PI3K/AKT signaling	[123]
Arjunolic acid	Saponin	Natural compound	IL-10 knockout mice	Inhibit intestinal epithelial cells apoptosis	[124]
Sophoricoside	Flavonoids	Natural compound	TNBS-induced colitis mice	Inhibit intestinal epithelial cells apoptosis by inhibiting PI3K/AKT signaling	[125]
Peiminine	Alkaloids	Natural compound	TNBS-induced colitis mice	Inhibit intestinal epithelial cells apoptosis by activating Nrf2/HO1 pathway	
swertiamarin	Iridoids	Natural compound	TNBS-induced colitis mice	Inhibit intestinal epithelial cells apoptosis by inhibiting PI3K/AKT signaling	[126]
isolongifolene	Turpentine	Natural compound	TNBS-induced colitis mice	Inhibit intestinal epithelial cells apoptosis by activating AMPK/PGC1α pathway	[127]
Necroptosis					
PP2		Synthetic compound	L929 mouse fibrosarcoma cells	Inhibit necroptosis by disrupting RIPK3 oligomerization	[132]
Pyroptosis					
Dz@MDSN		Synthetic compound	TNF^ΔARE^ mice	Inhibit pyroptosis in epithelial cells through ROS clearance	[133]
Tributyrin	glyceride	Synthetic compound	TNBS-induced colitis mice	Inhibit cGAS-STING-NLRP3 axis-mediated IECs pyroptosis	[134]
Ferroptosis					
Xue-Jie-San		Old drug	TNBS-induced colitis mice	Inhibit ferroptosis in IECs by inhibiting FGL1/NF-κB/STAT3 positive feedback loop	[135]

Andrographolide is the principal bioactive compound derived from Andrographis paniculata. It exerts anti-CD effects by four pathways: its antioxidant activity, suppressing STAT3 phosphorylation, promoting PD-L1-selective autophagy via P62/sequestosome-1 (SQSTM1) accumulation, and enhancing autophagic clearance via inhibiting NLRP3 inflammasome [92]. Glutamine, the most abundant free amino acid in the human body, serves as a crucial respiratory substrate and metabolic precursor for various cell types. Therapeutically, it demonstrates significant value in intestinal mucosal repair for managing enteropathies. Mechanistic studies reveal that ammonia generated during glutamine catabolism promotes autophagy activation. Furthermore, through coordinated regulation of the mTOR and MAPK signaling pathways, glutamine enhances IEC autophagy to inhibit apoptotic processes, thereby preserving intestinal barrier integrity [93]. Spermidine is a substrate for eukaryotic translation initiation factor 5A (eIF5A) hypusination modification. It facilitates autophagy-related gene translation to stimulate autophagy by inhibiting acetyltransferases and stimulating deacetylases [94, 95]. Spermidine may induce autophagy by inhibiting mTORC1 or triggering AMPK. Additionally, spermidine preserves key initiators of autophagosome formation by devitalizing caspase-3-mediated beclin1 cleavage [96]. Select phytochemical constituents and nutritional elements demonstrate therapeutic potential in CD through targeting autophagy. The inhibition of ROCK by AMA0825 could ameliorate obstruction induced by fibrosis in CD. It prevents and reverses intestinal fibrosis through suppressing myocardin-related transcription factor (MRTF) synthesis, attenuating p38 MAPK phosphorylation, and enhancing autophagic flux in fibroblasts [97]. Baicalin (BA) is a flavone glycoside isolated from Scutellaria baicalensis root. It ameliorates paracellular permeability by coordinated modulation of NF-KB activation, autophagic processes, and inflammatory cascades. BA enhances autophagic flux to lighten inflammation [98]. Total flavonoid aggregates (TFA) are a class of phytochemicals ubiquitously distributed in Plantae. It ameliorates intestinal fibrosis in CD by upregulation of p-AMPK expression and suppression of mTOR phosphorylation levels, which enhanced concurrent autophagy and apoptosis in intestinal fibroblasts [22]. Dietary supplementation with low molecular weight β-glucan (βGl) and high molecular weight β-glucan (βGh) exhibits temporally distinct modulation of colitis through autophagy-apoptosis interplay. βGl demonstrates predominant pro-apoptotic effects, while βGh shows superior autophagy potentiation. They collectively reduce colonic inflammation through pathway-specific temporal regulation [99]. The water-soluble derivative of chlorophyll (CHL) downregulates autophagic flux and lysosomal activity by activating the Akt/mTOR signaling axis. It attenuates colitis severity by suppressing pro-inflammatory cascades via this dual modulation [100].

Anakinra, an antagonist of human interleukin-1 (IL-1) receptor, is clinically employed in rheumatoid arthritis. Its capacity to restore autophagy levels in chronic granulomatous disease, attenuate IL-1-mediated inflammatory responses, and prevent TNBS-induced colitis suggests therapeutic potential for CD [101]. Sirolimus and everolimus exert immunosuppressive, antitumor, and antiviral effects exclusively through mTOR inhibition. mTOR inhibition suppresses T and B lymphocytes' proliferation upon cytokine signaling stimulation. Sirolimus is a macrolide immunosuppressant derived from the bacterium Streptomyces hygroscopicus. Everolimus, an autophagy inducer, is an analog of sirolimus used in preventing rejection following liver and kidney transplantation. In a retrospective study of pediatric patients with refractory IBD unresponsive to conventional therapies, 45% of UC patients and 100% of CD patients achieved clinical remission following sirolimus treatment [102]. P140, a selectively autophagy-targeting peptide, rectifies autophagic dysfunction in autoimmune and inflammatory disorders that rely on CMA mainly. P140 effectively modulates endogenous antigen processing and downstream deleterious pro-inflammatory events by suppressing hyperactivated autophagy [103]. Animal studies have shown that P140 treatment reduces the clinical and histological severity of colitis in CD-induced mice, suggesting it may serve as a valuable therapeutic target for IBD [104]. Azathioprine induces autophagy through modulation of the mTORC1 signaling pathway and stimulation of the unfolded protein response (UPR) sensor PERK. Its induction of autophagy correlates with enhanced clearance of AIEC and suppression of AIEC-driven TNFα upregulation [105]. Upadacitinib (UPA) can inhibit phosphorylation of JAK1 and STAT3, promote AMPK phosphorylation, and regulate autophagy-related biomarkers [106]. It clinically cures autoimmune diseases, such as ankylosing spondylitis, ulcerative colitis, and CD. Retinoic acid is a central metabolite of vitamin A, applied in dermatology and oncology. It can assuage intestinal inflammation. Retinoic acid ameliorates colitis by potentiating autophagy via inhibiting the PI3K/AKT/mTOR pathway [107]. In IL-10-deficient murine models, docosahexaenoic acid (DHA) mitigates CD symptoms by inhibiting the mTOR pathway and triggering autophagy [27].

Rapamycin alleviates disease activity index (DAI)scores and histological colitis severity in CD. Mechanistically, it downregulates expression of pro-inflammatory cytokines (TNF-α, IFN-γ, IL-17) and chemokines (CXCL-1, CXCL-2), and decreases intestinal and colonic permeability. In IL-10-deficient murine models, rapamycin restores mucosal barrier integrity [108]. In IL-10-deficient murine models, everolimus ameliorates CD-like ileitis [109], highlighting its potential anti-inflammatory mechanisms. γδIELs and their secretory factor API5 mitigate intestinal inflammation with ATG16L1 deficiency. In intestinal organoids of ATG16L1-deficient mice, it attenuates intestinal inflammation by restoring Paneth cell viability [110]. IL-10 deficiency correlated with very-early-onset IBD (VEO-IBD). Xue-Jie-San (XJS), a traditional Chinese medicine (TCM) prepared formulation, prevents early-stage CD-associated intestinal fibrosis by blockading dual Notch1 and fibrinogen-like protein 1(FGL1) pathway. Epithelial-mesenchymal transition (EMT) and endothelial-mesenchymal transition (EndoMT) are critical pathways in fibrogenesis initiation. XJS inhibits these pathways by provoking autophagy [23]. Sanguisorba officinalis is a traditional hemostatic agent, used in hematological disorders through thrombin-activating complex generation. In dextran sulfate sodium (DSS)-induced models, it demonstrates anti-colitis properties. It represents anti-inflammatory polarization in intestinal macrophages(Mφ) via potentiation of Atg7-dependent autophagy [111].

Regulating autophagy presents multiple therapeutic pathways for treating Crohn’s disease (CD), particularly through immune cells and intestinal epithelial cells (IECs). Andrographolide, glutamine, spermidine, and various phytochemicals enhance autophagic processes, modulate signaling pathways, and reduce inflammation, while agents like anakinra, sirolimus, and upadacitinib demonstrate clinical efficacy by restoring autophagy and inhibiting inflammatory responses.

### Anti-apoptotic drugs/modulators for CD treatment

As a critical component of the immune system, T cells are closely implicated in the pathogenesis and progression of CD. Notably, many therapeutic agents target surface molecules on T cells, further highlighting their potential as a key therapeutic target in CD management.

CD52 monoclonal antibody (CD52 mAb) directly targets cell surface CD52, effectively depleting mature lymphocytes through cytolytic effects. In IL-10(−/−) mice, D52 mAb alleviates colitis by restraining epithelial cell apoptosis via TNF-α/TNFR2 pathway [112]. Mesenchymal stem cells (MSCs) are used for cell therapy in various diseases. In inflamed and non-inflamed CD patient mucosa, co-culture with bone marrow-derived MSCs increases the apoptosis rate of T cells [113]. Adipose-derived mesenchymal stem cell (ADSC) alleviates TNBS-induced colitis by antagonizing TNBS induction, reducing expression of the classical Wnt pathway, increasing expression of the non-classical Wnt signaling pathway, and decreasing apoptosis rate and protein levels of cleaved caspase-3 [114]. Oridonin is an active component derived from Rabdosia rubescens. It alleviates TNBS-induced colitis through inhibiting NF-KB nuclear translocation, suppressing CD(+)T cell proliferation, upregulating lymphocyte apoptosis, and reducing colonic IFN-γ and IL-17 secretion [115]. Keratinocyte growth factor-2 (KGF-2), a multifunctional growth factor, stimulates the regeneration and reconstruction of the epidermis, dermis, and mucosa. STEA is a highly active human KGF-2 mutant. It promotes intestinal epithelial cell growth and relieves rats' colitis induced by TNBS through reducing serum levels of TNF-α, IL-1β, IFN-γ, and IL-6, increasing the cell proportion in the S phase, and inhibiting apoptosis [116]. SEW2871 is a selective agonist of the sphingosine-1-phosphate type 1 receptor. In IL-10 (−/−) mice, it alleviates colitis by reducing colonic lamina propria CD4+ T cells, lowering TNF-α and IFN-γ levels, inhibiting epithelial cell apoptosis, increasing tight junction protein (occludin and ZO-1) expression, and enhancing intestinal barrier function [117]. Dihydroartemisinin (DHA) is a derivative of artemisia annua with significant immunomodulatory activity. It alleviates TNBS-induced colitis via reducing the number of intestinal Th1 and Th17 cells, as well as Th9 and Th22 cells, increasing Treg cell numbers, inhibiting CD4+ T lymphocytes activation, and inducing their apoptosis [118].

In contrast to therapeutics targeting T cell surface molecules, a distinct class of drugs exerts direct effects on IECs to modulate CD pathogenesis.

Losartan is an AT1R blocker. It alleviates TNBS-induced colitis by inhibiting intestinal epithelial cell apoptosis via increasing the Bcl-2/Bax ratio and suppressing caspase-3 activation [119]. Adipose-derived mesenchymal stem cell (ADSC) alleviates TNBS-induced colitis by antagonizing TNBS induction, reducing expression of the classical Wnt pathway, increasing expression of the non-classical Wnt signaling pathway, and decreasing apoptosis rate and protein levels of cleaved caspase-3 [114]. Keratinocyte growth factor-2 (KGF-2), a multifunctional growth factor, stimulates the regeneration and reconstruction of the epidermis, dermis, and mucosa. STEA is a highly active human KGF-2 mutant. It promotes intestinal epithelial cell growth and relieves rats' colitis induced by TNBS through reducing serum levels of TNF-α, IL-1β, IFN-γ, and IL-6, increasing the cell proportion in the S phase, and inhibiting apoptosis [116]. Loureirin B (LB) is one of the most important chemical components and physiologically active ingredients of Dragon’s Blood. It ameliorates TNBS-induced colitis by stifling IL-6/STAT3/NF-κB signaling pathway activation and reducing intestinal epithelial cell apoptosis [120]. Poria cocos is a dried sclerotium parasitic on pine roots. Pachymic acid (PA) is one of the main bioactive components of Poria cocos. In CD mice, PA improves intestinal barrier damage and alleviates colitis by antagonizing intestinal epithelial cell apoptosis via inhibiting the PI3K/AKT signaling pathway [121]. Acetylcorynoline (Ace) is a major alkaloid component extracted from Corydalis bungeana. In TNBS treated CD model, it eases colitis by inhibiting intestinal epithelial cell apoptosis via inhibiting PI3K/AKT pathway activation [122]. Arjunolic acid (AA) is a saponin isolated from Cyclocarya paliurus with various biological activities, including antioxidant, antibacterial, and anti-inflammatory effects. In IL-10 (−/−) mice, AA mitigates colitis by inhibiting activation of the TLR4 pathway, reducing levels of Bax and Cleaved caspase-3, and suppressing intestinal epithelial cell apoptosis [123]. Sophoricoside (SOP) is an isoflavone glycoside isolated from Sophora japonica with anti-inflammatory, anticancer, and immunosuppressive effects. SOP protects the intestinal barrier and alleviates TNBS-induced colitis by reducing intestinal epithelial cell apoptosis via inhibiting the PI3K/AKT signaling pathway [124]. Peiminine (Pm) isolated from Fritillaria thunbergii.In CD mouse induced by TNBS, it improves colitis by inhibiting intestinal epithelial cell apoptosis via activating the Nrf2/HO1 pathway [125]. Swertiamarin (STM) has hypoglycemic, hypolipidemic, anti-rheumatic, and antioxidant effects. In the CD model, STM relieves colitis by suppressing intestinal epithelial cell apoptosis via inhibiting the activation of the PI3K/AKT signaling pathway [126]. Isolongifolene (ISO) is a tricyclic sesquiterpene isolated from Murraya koenigii. It mitigates colitis by reducing intestinal epithelial cell apoptosis via activating the AMPK/PGC1α signaling pathway [127]. Thymosin β4 (Tβ4) is a pro-lymphopoietic factor extracted from calf thymus in 1966. It is widely present in the human body and consists of 43 amino acids. Recombinant adeno-associated virus with Tβ4 (AAV-Tβ4) alleviates TNBS-induced colitis by reducing colonic TNF-α, IL-1β, and IL-10 levels, as well as inhibiting intestinal epithelial cell apoptosis [128]. Herbal cake-partitioned moxibustion (Moxi) is an indirect moxibustion method using moxa sticks. Moxi inhibits intestinal epithelial cell apoptosis induced by TNF-α, reduces intestinal epithelial permeability, and alleviates TNBS-induced colitis [129].

Natural killer (NK) cells, another crucial component of the immune system, have been implicated in CD pathogenesis through their dysregulated activity. 6-Mercaptopurine (6-MP) is a commonly used drug for CD treatment. It induces NK cell apoptosis by inhibiting the caspase-3/-9 inclusion pathway mediated by Rac1 [130].

A specific multi-fiber mixture (MF) matches the fiber content of a healthy diet. In IL-10 (−/−) mice, MF feeding ameliorates colitis by increasing expression and correcting distribution of occludin and ZO-1, reducing TNF-α/TNFR2 mRNA expression and epithelial cell apoptosis [131].

T cells play a critical role in the pathogenesis of Crohn’s Disease (CD), with various therapeutic agents targeting their surface molecules to manage the condition. Treatments such as CD52 monoclonal antibodies, mesenchymal stem cells, and specific compounds like Oridonin and KGF-2 have demonstrated efficacy in reducing T cell apoptosis and modulating inflammatory pathways, while other approaches focus on directly influencing intestinal epithelial cells to mitigate CD symptoms.

### Anti-necroptosis, pyroptosis, and ferroptosis drugs/modulators for CD treatment

PP2 is an inhibitor of SRC and RIPK3, which inhibits TNF-α-induced necroptosis without initiating apoptosis. It suppresses necroptosis by inhibiting MLKL phosphorylation and oligomerization via disrupting RIPK3 oligomerization [132].

Dz@MDSN is an oral pyroptosis nano-inhibitor. It targets macrophages at inflammatory sites and releases drugs in response to ROS loaded with TNF-α. It has potential for CD treatment by repairing the intestinal barrier and alleviating intestinal inflammation via inhibiting ROS-mediated intestinal epithelial cell pyroptosis [133].

Tributyrin is a neutral short-chain fatty acid triglyceride. It releases effective butyrate into cells by cell membrane diffusion and intracellular lipase metabolism. Tributyrin alleviates TNBS-induced colitis by inhibiting intestinal epithelial cell pyroptosis mediated by cGAS-STING-NLRP3 axis [134].

XJS alleviates TNBS-induced colitis by inhibiting intestinal epithelial cell ferroptosis via enhancing the SLC7A11/GSH/GPX4 pathway and inhibiting the FGL1/NF-κB/STAT3 pathway [135].

However, most of the drugs mentioned in the article (such as andrographolide, rapamycin, azathioprine, JAK inhibitors, etc.) have shown potential in mechanistic studies, but their clinical translation faces three major obstacles: ‘imprecise targeting, safety concerns, and difficulty in preventing drug resistance.’ Some drugs lack cell or tissue specificity, such as insufficient local drug concentration in the intestine, and are prone to systemic side effects. Regulating cell death pathways may further damage the intestinal barrier and exacerbate inflammation, and the gut microbiota can evade drug effects by altering host cell death pathways. Future research should focus on developing precise delivery systems, optimizing combination therapy strategies, conducting patient stratification and biomarker-guided approaches, as well as performing long-term safety monitoring and analysis of drug resistance mechanisms.

## Summary and future directions

Therapeutic resistance and suboptimal efficacy remain significant challenges in CD management. Resistance rates to biologic agents for CD treatment reach 30–40%. For instance, although anti-TNF antibodies are widely used in CD therapy, up to 40% of patients exhibit insufficient response. This may be associated with the degree of intestinal wall fibrosis and polymorphisms in the TLR4/MyD88 signaling pathway. As previously reported, reduced autophagy pathway activity in IBD patients with elevated Triggering receptor expressed on myeloid cells-1 (TREM-1) expression may represent one of the mechanisms underlying anti-TNF nonresponsiveness [136]. Another study demonstrates that autophagy modulation via mTORC1 and the unfolded protein response (UPR) may enhance the therapeutic efficacy of azathioprine in IBD [105], suggesting that autophagy activation can improve the effectiveness of anti-TNF antibodies and mitigate therapeutic resistance in CD. Therefore, autophagy induction represents a promising therapeutic strategy. However, research on autophagy in combination therapies remains underinvestigated. The role of autophagy in CD prognosis and disease progression has not been fully elucidated. By jointly targeting multiple cell death pathways such as autophagy, apoptosis, pyroptosis, necroptosis, and ferroptosis, it is expected to significantly improve the treatment effect of CD and overcome drug resistance through the synergistic regulation of intestinal inflammation, barrier function, and immune response.

Beyond autophagy, other forms of regulated cell death (RCD)—including apoptosis, pyroptosis, ferroptosis, and necroptosis—may also hold therapeutic potential for treatment-resistant CD. However, the mechanisms and clinical applications of these modalities require further investigation. Notably, while cell death may occasionally exacerbate CD progression, targeted induction of specific RCD pathways could conversely serve as an effective therapeutic strategy. This apparent paradox arises from their context-dependent roles: cell death in normal intestinal cells may disrupt mucosal homeostasis, thereby aggravating inflammation and CD pathogenesis, whereas selective elimination of damaged cells via these pathways might suppress disease progression and synergize with other therapies. Well-established agents with favorable safety profiles, such as azathioprine and upadacitinib, represent viable candidates for CD treatment. Crucially, safeguarding intestinal beneficial cells from aberrant cell death may emerge as a breakthrough direction in developing novel CD therapies.

**Fig. 6:**
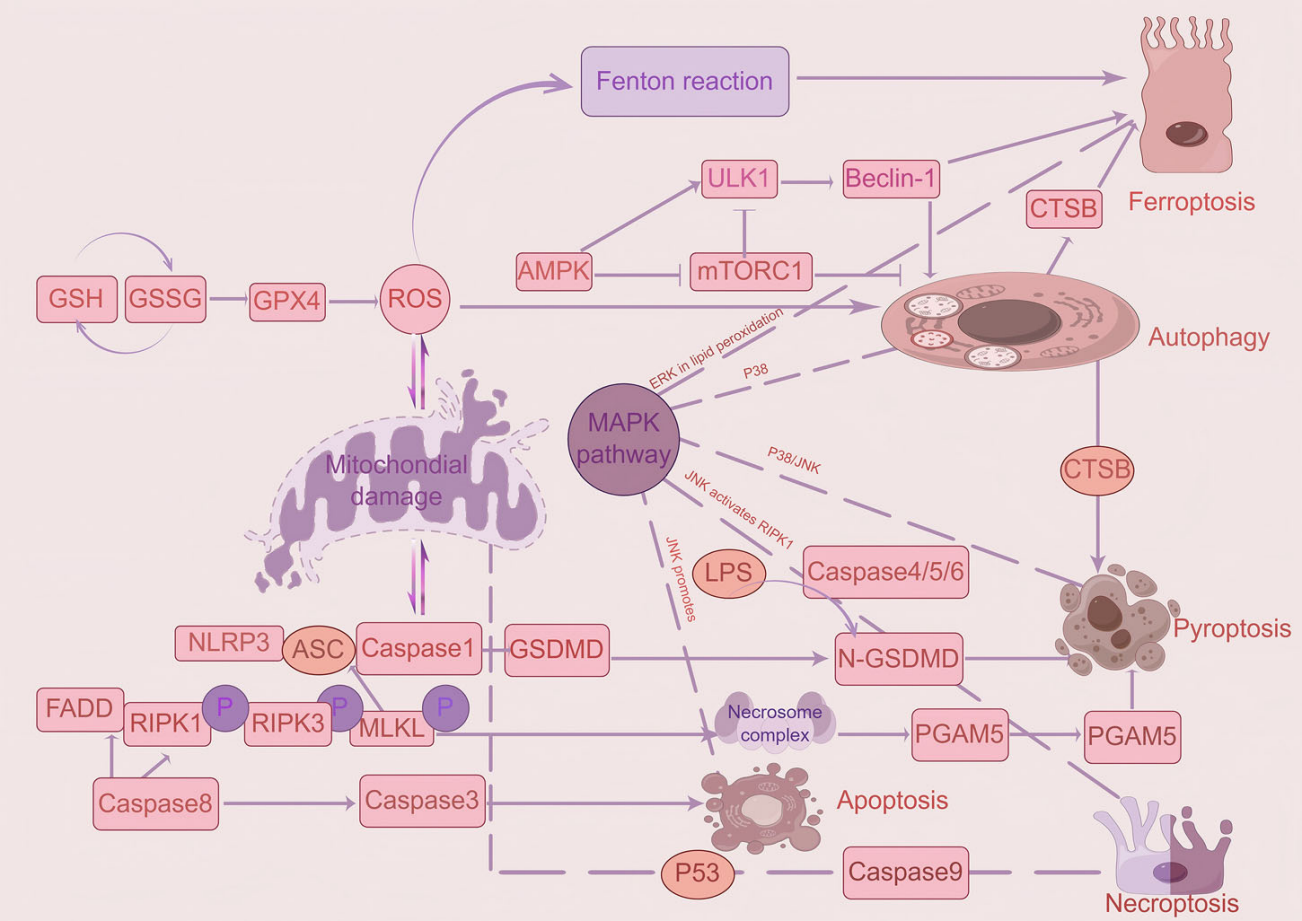
Crosstalk between ferroptosis, autophagy, apoptosis, necroptosis, and pyroptosis. Among the five modes of cell death, autophagy demonstrates an association with ferroptosis and pyroptosis via CTSB. ROS production generally activates autophagy directly while inducing mitochondrial damage, which subsequently triggers pyroptosis through the Caspase-1 pathway and initiates necroptosis via the P53 pathway. Concurrently, ROS-dependent Fenton reaction contributes to ferroptosis activation. Caspase-8 mediates pyroptosis through the RIPK pathway while activating apoptosis via caspase-3. The MAPK pathway manifests regulatory effects across all five death modalities: ERK phosphorylation within this pathway directly modulates ferroptosis through lipid peroxidation regulation, P38 signaling governs autophagy, and the P38/JNK axis participates in pyroptosis. Additionally, JNK signaling facilitates necroptosis through RIPK1 activation while promoting apoptosis.

Different forms of cell death may exhibit functional crosstalk, with ferroptosis, necroptosis, pyroptosis, and oxeiptosis all requiring the generation of ROS (Fig. 6 and Table 2). Recent studies identify interactions between autophagy and pyroptosis [137], as well as an apoptosis-pyroptosis molecular switch in which caspase-8 cleaves Gasdermin D (GSDMD) at the Asp88 site to generate functional fragments that determine inflammatory intensity (NLRP3 activation threshold). These findings suggest that therapeutic agents targeting individual cell death modalities may yield suboptimal clinical outcomes. Consequently, further exploration of these intricate mechanistic interrelationships is critical to comprehensively elucidate their roles in CD.

**Table 2:** Crosstalk between ferroptosis, autophagy, apoptosis, necroptosis, Oxeiptosis, and pyroptosis.

Molecular/Pathway	Ferroptosis	Pyroptosis	Autophagy	Apoptosis	Necroptosis	Oxeiptosis
ROS	Core Drivers	Secondary trigger	Indirect regulation	Mitochondrial pathway triggering	Promote necrotic signals	Directly triggered
Caspases	Not directly involved	Caspase-1/4/5/11	Caspase-3 (specific)	Caspase-3/8/9	Independent	May participate
Bcl-2 family	Indirect regulation	Not related	Interacting with Beclin-1	Core regulation	Not related	May be relevant
NF-κB	Inflammation amplification	Inflammatory core	Indirect regulation	Bidirectional regulation	Inflammatory association	May participate
MAPK pathway	ERK participates in lipid peroxidation	P38/JNK regulates inflammation	P38 regulates autophagy	JNK promotes apoptosis	JNK activates RIPK1	Possible activation
Mitochondrial damage	Lipid peroxidation	Minor role	Mitophagy	Membrane permeability	ROS promotes necrosis	Direct Damage

Above all, a bidirectional, self-perpetuating cycle exists between CD and cell death, wherein CD pathogenesis triggers multiple forms of regulated cell death (RCD), while dysregulated RCD pathways exacerbate mucosal barrier dysfunction and chronic inflammation. Modulating these pathways, including apoptosis, autophagy, pyroptosis, ferroptosis, and necroptosis, offers therapeutic potential to restore mucosal homeostasis and mitigate CD progression. This review systematically dissects the multifaceted roles of cell death in CD across pathogenesis, disease stratification, and treatment response, integrating pre-clinical models, clinical correlations, and therapeutic targets. By synthesizing mechanistic insights from molecular signaling cascades to translational applications, we highlight opportunities to leverage RCD modulators as novel therapeutic strategies. Future research should focus on the following directions: First, to conduct comprehensive and in-depth validation of the efficacy and safety of these therapeutic targets within patient populations; second, to actively explore the combined application of different drugs or therapies to achieve synergistic effects; and third, to delve into the mechanisms of interaction between different types of cell death at various stages of the disease. Through these studies, it is hoped that more precise and efficient strategies for the treatment of Crohn’s disease can be developed.
